# Triple Viral Infections in Advanced Breast Cancer: Insights from a Three-Case Report and Literature Review

**DOI:** 10.3390/diagnostics15010051

**Published:** 2024-12-28

**Authors:** Ashraf I. Khasawneh, Nisreen Himsawi, Mohammed Alorjani, Hadeel Al-Momani, Uruk Shahin, Ashraf Sammour, Tareq Saleh, Hafez Al-Momani, Rame Khasawneh, Sofian Al Shboul

**Affiliations:** 1 Department of Microbiology, Pathology, and Forensic Medicine, Faculty of Medicine, The Hashemite University, Zarqa 13133, Jordan; 2 Department of Pathology and Microbiology, Faculty of Medicine, Jordan University of Science and Technology, Irbid 22110, Jordan; 3 Department of Pharmacology and Public Health, Faculty of Medicine, The Hashemite University, Zarqa 13133, Jordan; 4 Department of Anatomy, Physiology & Biochemistry, Faculty of Medicine, The Hashemite University, Zarqa 13133, Jordan; 5 King Hussein Medical Center, Royal Medical Services, Amman 11942, Jordan

**Keywords:** breast cancer, RT-PCR, Jordan, CMV, BLV, MMTV

## Abstract

**Background and clinical significance:** Viral infections are typically considered contributing or secondary factors in the development of breast cancer. **Case presentation**: This case report presents three instances of advanced breast cancer associated with triple viral infections. Case 1 involves a 78-year-old woman diagnosed with grade 2 invasive ductal carcinoma positive for HPV-18, CMV, and BLV. Case 2 describes a 39-year-old woman with grade 2 invasive ductal carcinoma, positive for HPV-16, CMV, and BLV. Case 3 is a 52-year-old woman with grade 3 invasive ductal carcinoma, positive for HPV-16, BLV, and MMTV. These cases suggest a possible correlation between viral co-infections and breast cancer aggressiveness, posing new questions about the role of viral infections in cancer development and prognosis. **Conclusions:** The findings contribute to growing evidence that viral infections may influence the progression and therapeutic response of breast cancer, warranting further investigation into targeted preventive measures including vaccinations.

## 1. Introduction

Viral infections are increasingly recognized as primary contributors to cancer development, with oncogenic viruses implicated in approximately 12% of all human cancers worldwide [1]. Several viruses are known to have potent oncogenic potential, with well-documented associations between Human Papilloma Virus (HPV) and cervical cancer, Epstein–Barr Virus (EBV) and Burkitt’s lymphoma, and Hepatitis B Virus (HBV) with hepatocellular carcinoma [2]. These viruses not only initiate carcinogenesis but may also exacerbate disease aggressiveness, influence tumor characteristics, and affect patient prognosis [2]. Importantly, cancers associated with viral infections are more commonly reported in developing regions and areas with high endemicity of these viruses, underscoring the influence of both geographic and socioeconomic factors on viral carcinogenesis [1].

Breast cancer, the most prevalent cancer among women globally, has been increasingly linked to viral infections, particularly in recent years as molecular techniques have evolved to detect viral genomes within breast tissue. Multiple viruses, including HPV, EBV, Cytomegalovirus (CMV), Bovine Leukemia Virus (BLV), and Mouse Mammary Tumor Virus (MMTV), have been identified as potential contributors to breast cancer pathogenesis [3,4]. The detection of these viruses in breast cancer tissues has spurred considerable interest in understanding how chronic or latent viral infections could play a role in the onset and progression of breast cancer. Though the causal link remains under investigation, the presence of viral DNA within breast tumors raises critical questions about viral oncogenesis and the potential for specific viruses to serve as co-factors in breast cancer development.

Establishing a clear link between viral infections and breast cancer could pave the way for preventive vaccines and targeted antiviral therapies, potentially revolutionizing breast cancer management. Moreover, a deeper understanding of how viral infections influence breast cancer development might enable more personalized treatment approaches, allowing for the identification of high-risk patients with specific viral co-infections who could benefit from customized therapeutic strategies. While most studies have focused on the role of individual viruses in breast cancer, the contribution of multiple concurrent infections remains largely unexplored. In this report, we present three cases of advanced breast cancer, each involving a unique combination of three viral infections—HPV, BLV, and either CMV or MMTV. These cases represent an intriguing area of study, as they suggest a potential synergy between multiple viruses in contributing to breast cancer pathogenesis. The co-existence of these infections could imply a complex interplay of viral and host factors that together foster an environment conducive to malignancy. This unique presentation underscores the importance of investigating viral co-infections as a possible factor in breast cancer development and progression, and it highlights the need for further research to determine whether these viral interactions may influence cancer prognosis, aggressiveness, or resistance to therapy.

Our study adds to a growing body of evidence that suggests viral co-infections may be more prevalent in breast cancer patients than previously recognized. The regional and international variability in viral infection rates and combinations observed in breast cancer cases highlights the need for a global perspective on viral oncogenesis research. By studying Jordanian breast cancer patients, a relatively understudied population in viral-oncogenesis research, we aim to contribute to the diversity of existing data and provide insights into how viral co-infections may affect breast cancer patients across different demographics. This report provides new insights into the potential role of multiple viral infections in breast cancer through the presentation of three unique cases. These cases suggest that complex interactions between viruses, along with patient-specific risk factors, could contribute to the pathophysiology of breast cancer. The findings highlight the importance of considering viral co-infections as part of a comprehensive approach to breast cancer etiology, with implications for future research into cancer prevention, diagnosis, and treatment.

## 2. Case Presentation

### 2.1. Case 1

A 78-year-old woman presented with a progressively enlarging right breast mass over six months. She had a history of a cardiac catheterization procedure (no stent placement) and no significant family history of cancer or genetic predispositions. Upon initial evaluation, a low-dose non-enhanced CT scan and a full-body PET scan revealed a mass in the upper outer quadrant of the right breast measuring 3.6 × 2.2 × 2 cm. No evidence of lymph node involvement or distant metastases was detected, confirming a localized disease.

**Figure 1:**
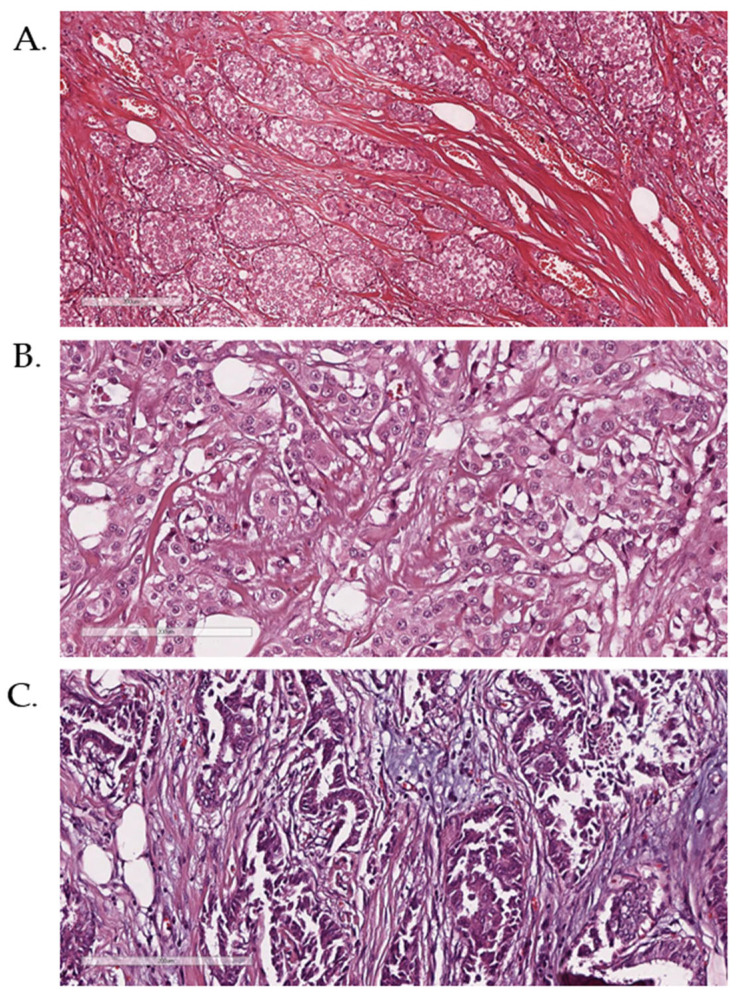
**H&E histopathological images demonstrating breast cancer diagnosis in the 3 cases (A–C)**. Figure scale is 200 µm.

A true-cut biopsy of the mass confirmed Stage II Invasive Ductal Carcinoma (IDC) that was not otherwise specified (NOS) (Figure 1A). Immunohistochemistry (IHC) analysis demonstrated hormone receptor positivity, with the tumor being Estrogen Receptor-positive (ER+) and Progesterone Receptor-positive (PR+) but Human Epidermal Growth Factor Receptor 2-negative (HER2-). These findings classified the tumor as the luminal A subtype, indicating a favorable prognosis and hormone sensitivity. Following the diagnosis, the patient underwent a total mastectomy without adjuvant chemotherapy and was placed on long-term endocrine therapy with an aromatase inhibitor.

**Figure 2:**
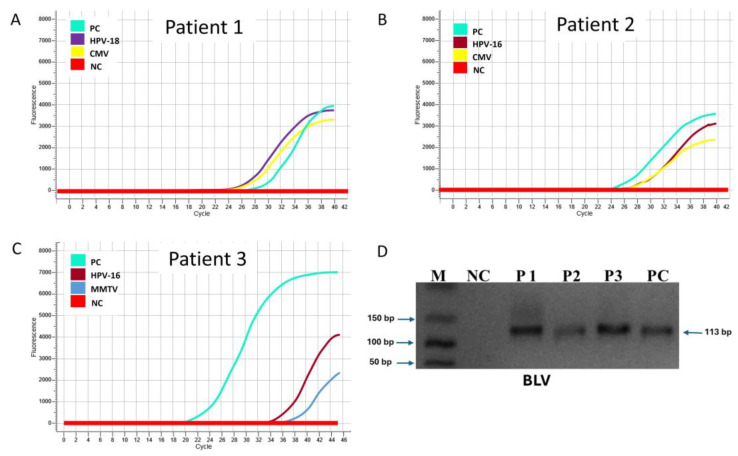
**RT-PCR amplification curves and agarose gel image showing the detection of probable oncogenic viruses associated with BC in our patients.** In patient 1 (**A**), HPV-18 and CMV were detected, while patient 2 (**B**) showed the presence of HPV-16 and CMV. Patient 3 (**C**) was positive for HPV-16 and MMTV. BLV was detected in all three patients (**D**). RNA extraction was performed using the RNeasy FFPE Kit (QIAGEN, Hilden, Germany) according to the manufacturer’s protocol. The extraction included incubation with Buffer PKD and Proteinase K, followed by DNase treatment and RNA purification using spin columns. Extracted RNA was converted to cDNA using the QuantiTect Reverse Transcription Kit (QIAGEN, Hilden, Germany). For HPV detection, the REALQUALITY RQ-Multi HPV kit (AB ANALITICA, Padova, Italy) was employed, enabling the identification of 28 HPV genotypes, including high- and low-risk types. EBV, CMV, MMTV, and BLV detection was conducted using specific primers targeting the EBNA2 gene, GB region, env gene, and tax gene, respectively. The PCR products were visualized on a 2% agarose gel using a UV documentation system. Quantitative RT-PCR was performed with the QuantiTect SYBR Green PCR Kit (QIAGEN, Hilden, Germany) to amplify target DNA.

The formalin-fixed paraffin-embedded (FFPE) tissue block of the tumor underwent deparaffinization and RNA extraction. Real-time polymerase chain reaction (PCR) testing revealed co-infection with three oncogenic viruses: Human Papillomavirus (HPV) type 18, Cytomegalovirus (CMV), and Bovine Leukemia Virus (BLV) (Figure 2A,D). A high-risk HPV subtype known for its oncogenic potential, HPV-18 is associated with persistent infection and the promotion of cellular dysregulation through the E6 and E7 oncoproteins, which inhibit p53 and RB tumor suppressor pathways. Its presence in breast tissue suggests a potential contributory role in carcinogenesis. Known to induce a pro-inflammatory tumor microenvironment (TME), CMV has been implicated in the modulation of immune responses and angiogenesis, potentially accelerating tumor growth. A retrovirus primarily associated with bovine lymphomas, BLV has been detected in human breast cancer tissues. Its integration into the genome and associated chronic inflammation may contribute to oncogenesis, though this remains controversial.

The patient’s immunological response to the viral infections was evaluated using cytokine profiling from blood plasma. The analysis revealed elevated levels of pro-inflammatory cytokines, including interleukin-6 (IL-6) and tumor necrosis factor-alpha (TNF-α), which are often linked to chronic inflammation and tumor progression. No clinical signs of systemic immune suppression, such as neutropenia or lymphopenia, were noted.

This case illustrates the complex interplay between viral infections and breast cancer pathophysiology. The presence of three oncogenic viruses raises questions about synergistic effects in promoting tumor initiation and progression. While a direct causal link cannot be established, this finding underscores the need for larger studies to explore the potential mechanisms by which multiple viral co-infections may influence breast cancer biology. The patient remains in remission under endocrine therapy with no recurrence of the disease during a follow-up period of 12 months. Further monitoring and immunological assessments are planned to evaluate long-term outcomes and the role of latent viral infections in recurrence risk.

### 2.2. Case 2

A 42-year-old married woman presented with a progressively enlarging right-sided breast mass, initially noted 18 months ago. Over the past year, she experienced a significant weight loss of approximately 8 kg. She reported no remarkable family history of cancer, but her past medical history included type 2 diabetes mellitus, controlled with oral hypoglycemic agents. The patient had a long-term history of tobacco use, smoking one and a half packs per day for the past 25 years.

Initial imaging included a low-dose non-enhanced CT scan followed by a whole-body PET scan, which revealed a 4.2 × 3.8 cm hypermetabolic lesion in the upper outer quadrant of the right breast. The scan also identified small, hypermetabolic axillary lymph nodes suggestive of metastasis and a subcentimeter mediastinal node with equivocal uptake. A modified radical mastectomy with axillary lymph node dissection was performed. Histopathological evaluation confirmed Grade 3 IDC (NOS), which tested positive for ER+ and PR+ receptors but HER2-, consistent with a luminal B subtype (Figure 1B). Axillary lymph node analysis revealed metastatic deposits in two out of 12 dissected nodes.

The patient underwent adjuvant chemotherapy with dose-dense doxorubicin and cyclophosphamide, followed by weekly paclitaxel combined with trastuzumab, given the HER2-positive nature of the tumor. She also received external beam radiation therapy to the chest wall and axilla due to nodal involvement. Hormonal therapy with tamoxifen was initiated, given the hormone receptor-positive status of the tumor.

FFPE tissue sections of the tumor were analyzed for viral infections using real-time PCR. The results revealed co-infection with HPV-16, EBV, and CMV (Figure 2B,D). A high-risk oncogenic subtype, HPV-16, is known for its strong association with carcinogenesis through E6- and E7-mediated disruption of tumor suppressor genes. The presence of EBV in breast cancer tissue highlights its potential role in oncogenesis by promoting immune evasion and inflammatory pathways. As seen in Case 1, CMV was detected and is thought to contribute to an immunosuppressive tumor microenvironment and enhance angiogenesis.

Cytokine profiling from serum samples demonstrated elevated levels of interleukin-10 (IL-10) and transforming growth factor-beta (TGF-β), both of which are indicative of a tumor-promoting immunosuppressive state. Interestingly, there was no evidence of systemic immune dysfunction, such as lymphopenia, but localized inflammation was evident. At a 12-month follow-up, the patient remained in clinical remission with no radiological evidence of recurrence. However, close monitoring was advised, given the aggressive nature of the tumor and the presence of multiple viral co-infections.

### 2.3. Case 3

A 54-year-old female presented with a three-month history of a rapidly enlarging, painful, and erythematous right breast mass. Her medical history was significant for type 2 diabetes and hypertension, but she reported no remarkable surgical or family history of cancer. She denied any systemic symptoms such as weight loss or fever. Initial imaging with a low-dose non-enhanced CT scan followed by a whole-body PET scan revealed a large, hypermetabolic right breast mass measuring 10.5 × 9.8 × 5.2 cm. The scan also demonstrated extensive hypermetabolic nodal involvement, including right axillary, sub-pectoral, infraclavicular, and mediastinal lymph nodes. Additionally, there was a hypermetabolic osteolytic lesion in the T10 vertebra, consistent with bone metastasis.

A true-cut biopsy was performed, which confirmed Grade 3 IDC (NOS). The tumor was hormone receptor-negative for Progesterone Receptor (PR-) but tested positive for Estrogen Receptor (ER+) and Human Epidermal Growth Factor Receptor 2 (HER2+) (Figure 1C). The patient was treated with neoadjuvant chemotherapy consisting of dose-dense doxorubicin and cyclophosphamide, followed by paclitaxel combined with trastuzumab (Herceptin). After achieving a partial response, she underwent a right partial mastectomy and axillary lymph node dissection. Post-surgery, she continued trastuzumab and received radiation therapy targeting the chest wall and metastatic lesion in the T10 vertebra.

In May 2022, the patient presented with symptoms of headache, nausea, vomiting, and difficulty walking, which progressed to significant lower limb weakness and loss of balance. Brain MRI with contrast revealed three metastatic lesions in the right cerebellum and left frontal lobe, with associated vasogenic edema. She was treated with high-dose corticosteroids and whole-brain radiotherapy. In October 2022, the patient was admitted with symptoms of severe dyspnea and fever. A chest CT scan revealed extensive consolidation in the left lower lung consistent with bacterial pneumonia. Despite aggressive antibiotic therapy, the patient’s condition deteriorated, and she succumbed to respiratory failure.

Molecular analysis of the FFPE tumor tissue using real-time PCR identified triple viral infections (Figure 2C,D). HPV-16 is a high-risk oncogenic subtype strongly associated with tumorigenesis. BLV has been implicated in promoting genomic instability and a pro-inflammatory tumor microenvironment. The detection of MMTV-like sequences in human breast cancer raises concerns about its potential role in oncogenesis, though its contribution remains controversial. Serum cytokine analysis demonstrated elevated levels of pro-inflammatory markers, including IL-6 and TNF-α, indicative of a systemic inflammatory response. The co-existence of three oncogenic viruses in the tumor tissue highlights the potential multifactorial contributions to tumor progression and metastatic behavior.

The literature describes multiple studies, which explored the link between multiple viral infections and BC. However, there is limited research specifically examining the impact of viral co-infections on the progression of breast cancer, particularly in advanced stages. Our cases highlight the potential role of viral co-infections in the progression and pathophysiology of breast cancer. They emphasize the complexity of these infections in cancer patients, suggesting that viral interactions may influence disease outcomes, immune responses, or treatment efficacy. These cases also underline the need for further investigation into the molecular mechanisms linking viral infections to cancer development and the importance of considering viral co-infections in cancer diagnostics and management.

## 3. Discussion and Systematic Review

The association between viral infections and cancer development has opened new avenues for both treatment and prevention strategies. These approaches include targeting the infectious agents and infected cells or developing vaccines that could prevent initial infections, potentially leading to decreased cancer rates associated with specific viruses. For instance, the success of the HPV vaccine in reducing cervical cancer rates and the effectiveness of the HBV vaccine in preventing liver cancer are prime examples of the potential for this approach in cancer prevention. For breast cancer, however, the role of viral infections is complex and is generally viewed as a contributing factor rather than a primary cause [5]. Breast cancer is a multifactorial disease, and the interplay between inherent risk factors—such as genetics, lifestyle, and environmental exposures—and viral infections appears to create a conducive environment for malignancy [6]. This complexity underscores the need for a refined approach to understanding breast cancer etiology and viral involvement.

We conducted a systematic review of the literature to explore the link between multiple viral infections and breast cancer, analyzing recent publications from the past ten years to identify the most common etiological factors associated with BC pathogenesis and progression.

For this review, we followed the Preferred Reporting Items for Systematic Reviews and Meta-Analyses (PRISMA) guidelines. We searched the PubMed database using the keywords “breast cancer”, “virus infection”, and one of the following terms at a time: “BLV”, “CMV”, “EBV”, “HPV”, or “MMTV”. We considered original articles and case reports only. Title and abstract screening were performed independently by two reviewers, with a third acting as a mediator when discrepancies arose.

**Figure 3:**
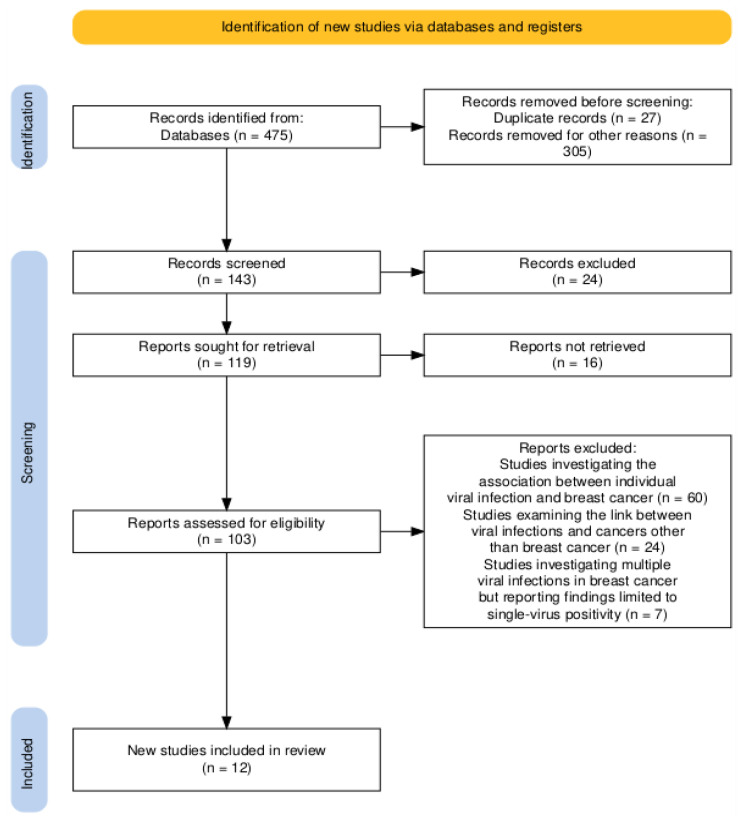
**PRISMA flowchart of the study selection process for the systematic review of viral infections and breast cancer**.

Inclusion criteria for the review were the following: full-text, English-language articles discussing multiple viral infections linked to breast cancer. We excluded articles that were not in English, studies focusing on single viral infections, and those related to viral infections in cancers other than breast cancer. The search identified 475 articles published between January 2015 and November 2024. After removing duplicates and screening titles and abstracts, 108 studies were further evaluated for eligibility. Ultimately, 12 articles met the inclusion criteria and were included in the review (Table S1). The selection process of the included studies is illustrated in the flow diagram in Figure 3.

### 3.1. Mechanisms of Viral Oncogenesis and Immune Response in Breast Cancer

Oncogenic viruses, including HPV, BLV, and EBV, have been well-documented for their ability to interfere with host cell mechanisms and promote cancerous transformations [7]. Viruses can contribute to cancer development through several mechanisms: integration of viral DNA into the host genome, which can lead to mutations; inhibition of tumor suppressor pathways; and immune modulation, which allows infected cells to evade detection and destruction by the immune system [8]. High-risk HPV types (e.g., HPV16 and HPV18) express viral oncoproteins like E6 and E7, which inactivate tumor suppressors p53 and Rb, leading to uncontrolled cellular division and potential malignancy [9].

Interestingly, our cases suggest that an asymptomatic viral infection may remain dormant for extended periods and reactivate during periods of immunosuppression, as observed in Case 1, where surgery-induced immune compromise appeared to trigger viral reactivation and potentially contributed to unchecked cellular division. This pattern raises critical questions about the impact of chronic or latent infections on breast cancer pathogenesis, particularly in patients who may already possess inherent risk factors, such as nulliparity, advanced age, smoking, or hypertension. While no single factor, whether viral or lifestyle-related, appears sufficient to induce breast cancer, their synergistic effects create an environment that enhances cancer development. For example, HPV’s oncogenic effects are amplified in the presence of risk factors like smoking, which increases DNA damage [10]. Similarly, EBV’s activation of cell proliferation and immune evasion pathways may be more pronounced in individuals already experiencing cellular stress due to age or hypertension [11]. Infections with MMTV, BLV, and CMV further contribute to this complex environment, triggering inflammation, immune dysregulation, and DNA damage, which collectively promote malignancy.

**Figure 4:**
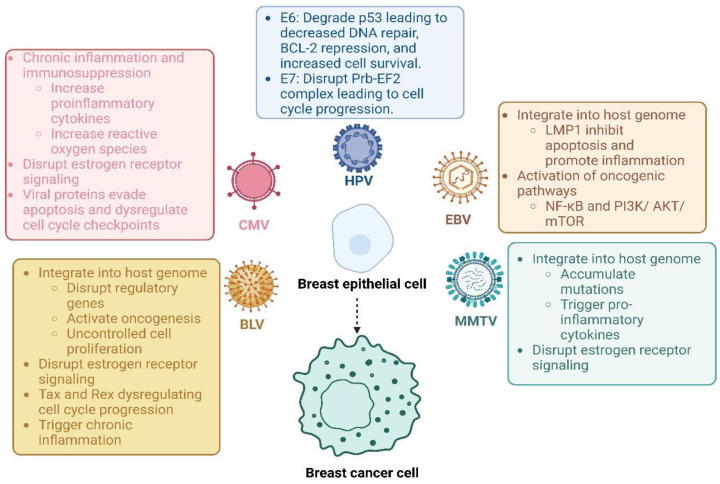
**Probable Mechanisms of viral oncogenesis in breast cancer**. Viral oncogenesis in breast cancer likely involves multiple mechanisms, with each virus contributing to tumorigenic processes in distinct ways. HPV promotes malignancy by disrupting tumor-suppressor pathways; its E6 and E7 proteins inactivate p53 and the pRB-E2F complex, leading to unchecked cell growth and impaired DNA repair. EBV influences breast cells by activating the HER2/HER3 pathways and NF-κB signaling via its LMP1 protein, promoting cell proliferation and immune evasion. MMTV drives inflammatory responses through superantigens, creating a pro-inflammatory microenvironment, while BLV’s Tax protein activates NF-κB and disrupts DNA repair, supporting continuous cell division and mutation. CMV contributes to cancer risk by inducing chronic inflammation and activating telomerase, which aids cellular immortality. These viral effects may be compounded in patients with additional risk factors, such as smoking, hypertension, or aging, which further compromise immune defenses and enhance cancer susceptibility through cumulative cellular stress, immune dysregulation, and DNA damage.

These viral infections, each with distinct mechanisms of oncogenesis, interact with risk factors to create a multifactorial pathogenesis of breast cancer. MMTV is thought to activate immune responses that drive inflammation through superantigens, potentially contributing to a microenvironment supportive of cancer [12]. BLV’s ability to inhibit DNA repair and activate continuous cell division aligns with the patterns seen in breast cancer, especially in individuals with impaired DNA repair mechanisms [13]. CMV’s ability to induce chronic inflammation and telomerase activation further supports cellular immortality, particularly in patients with risk factors that affect immune responses and cellular aging [14]. In sum, chronic infections and pre-existing risk factors work together, compounding the risk of breast cancer through a combination of cellular stress, inflammation, immune dysregulation, and DNA damage (Figure 4).

### 3.2. Regional Variability and Frequency of Viral Co-Infections in Breast Cancer

The frequency of viral co-infections, as demonstrated in our study, mirrors findings from other regions yet exhibits significant variability. In Jordan, Al-Hamad et al. reported multiple viral infections in 5.7% of breast cancer patients, while higher rates were observed in studies from Lebanon (29%), Qatar (47%), and Syria (32%) involving high-risk HPV and EBV co-infections [15,16,17,18]. Such regional disparities underscore the importance of considering local epidemiological factors, including variations in viral prevalence and host susceptibility, when assessing the role of viral infections in breast cancer. Moreover, our findings of triple viral co-infections (HPV, BLV, and either CMV or MMTV) align with a growing body of evidence supporting viral involvement in breast cancer. For example, Metwally et al. documented triple viral infections (HPV, EBV, and MMTV) in an Egyptian cohort, indicating that co-infection patterns may be more prevalent in certain geographic regions and ethnic groups [19]. Similarly, El-Shinawi et al. reported significantly higher rates of co-infections such as HPV-16/CMV/HHV-8 in inflammatory breast cancer (IBC), linking these patterns to increased tumor proliferation and aggressiveness through Ki-67 overexpression [20].

The notable presence of BLV and MMTV in our cases, particularly in conjunction with HPV, highlights the complexity of viral interactions and their potential influence on tumor behavior, aggressiveness, and therapeutic resistance. Interestingly, Cases 1 and 2 in our study, which showed co-infections with HPV, BLV, and CMV, were classified as grade 2 tumors, while Case 3 presented a grade 3 tumor with HPV, BLV, and MMTV co-infections. HPV and EBV were the most commonly reported co-infections across multiple studies, including those from Syria, Qatar, and Lebanon. Co-infections involving CMV were also reported in Egypt and Peru [20,21]. Less frequently observed co-infections included MMTV and BLV (1.9%) in Jordan and EBV and MMTV (6%) in Pakistan [3,22]. However, other studies, such as those by Al Hamad et al. and Gupta et al., did not find significant correlations between viral co-infections and clinicopathological features, reflecting the need for more targeted research to clarify these interactions [15,23,24].

Taken together, these findings emphasize the importance of understanding the molecular mechanisms of viral co-infections in breast cancer. Co-infections may not only drive tumor progression but also contribute to therapeutic resistance, particularly in cases involving less-studied viruses like BLV and MMTV. Such insights could have significant implications for breast cancer diagnostics, treatment strategies, and epidemiological surveillance.

### 3.3. Potential Impact of Viral Co-Infections on Prognosis and Cancer Aggressiveness

The presence of multiple viral infections in breast cancer cases raises several critical questions about their impact on prognosis, cancer aggressiveness, resistance to therapy, and recurrence. An intriguing hypothesis is that the co-existence of multiple viral infections could stimulate a heightened immune response, potentially mitigating cancer progression. Viral infections may induce chronic inflammation, alter immune responses, or create an immune-suppressive microenvironment, all of which could influence cancer behavior [25]. However, the relationship between chronic inflammation, immune modulation, and viral persistence in cancerous tissues remains poorly understood and warrants further exploration.

In some cancers, viral co-infections have been shown to enhance each other’s oncogenic effects, potentially leading to more aggressive disease [26]. It remains speculative whether this phenomenon is applicable to breast cancer. The current literature, while suggestive, does not provide definitive answers regarding the prognostic implications of co-infections in breast cancer. For example, our cases indicated that co-infections were associated with higher tumor grades, but without large-scale data, it is challenging to determine whether these findings are broadly applicable or are unique to certain patient populations or tumor subtypes.

### 3.4. Implications for Breast Cancer Prevention and Treatment Strategies

The identification of viral infections in breast cancer opens up new potential strategies for treatment and prevention. Similar to the role of the HPV vaccine in preventing cervical cancer and the HBV vaccine in preventing liver cancer, the development of vaccines or antiviral therapies targeting breast cancer-associated viruses could offer a preventive measure for high-risk populations. Currently, vaccines for viruses such as HPV have shown success in reducing virus-associated cancers, suggesting that similar strategies might be beneficial for breast cancer if specific viral associations can be conclusively demonstrated.

To build on this, existing and emerging preventive and therapeutic strategies can serve as models. For instance, vaccines like the HPV vaccine have effectively reduced the incidence of cervical and other virus-associated cancers, offering a proof of concept for targeting oncogenic viruses. Oncolytic virus-based immunotherapies are another promising avenue, currently under investigation in clinical trials for breast cancer [27,28]. These therapies leverage the virus’s ability to selectively infect and destroy cancer cells while simultaneously stimulating the host immune system to target malignancies.

Future research should aim to clarify whether viral co-infections are merely bystanders in the carcinogenic process or play a more direct role in oncogenesis. Such studies should investigate the feasibility of targeted antiviral therapies or immunotherapies for breast cancer patients who test positive for oncogenic viruses. Immunotherapy, which has shown promise in other cancers, may have a role in managing virus-associated breast cancer by stimulating the immune system to recognize and eliminate infected cells. However, more comprehensive studies are needed to explore the utility of these therapies in breast cancer and determine if viral infections can serve as reliable biomarkers for patient prognosis or therapeutic response.

### 3.5. Limitations and Future Research Directions

While our case series contributes valuable insights into the role of viral co-infections in breast cancer, it has certain limitations. Firstly, the limited sample size may not be generalizable across all populations. Secondly, the complexity of viral co-infections and their interactions with host factors complicates the interpretation of these findings. Finally, the lack of standardized protocols for detecting and confirming viral infections in breast cancer samples would lead to potential discrepancies in viral prevalence across studies.

Moreover, while our study provides preliminary evidence of distinct cytokine profiles among the cases (elevated IL-6 and TNF-alpha in patients 1 and 3 and elevated IL-10 and TGF-beta in patient 2), the limited dataset does not allow for conclusive correlations between these immune markers, viral infections, and breast cancer pathogenesis. These cytokine patterns suggest differing immune responses, with IL-6 and TNF-alpha indicative of a pro-inflammatory environment, while IL-10 and TGF-beta imply an immunosuppressive microenvironment. However, the cross-sectional nature of our observations restricts our ability to establish causality or explore temporal dynamics.

Despite these limitations, the emerging patterns observed in our study and others underscore the need for larger, multicenter studies to better understand the role of viral infections in breast cancer. Given the significant variation in viral co-infection rates across regions, an internationally collaborative research effort is essential for fully elucidating the impact of these viruses on breast cancer. Such efforts could lead to the establishment of globally standardized testing and reporting protocols, enabling more accurate comparisons across studies and populations. Future research should also aim to integrate longitudinal data and mechanistic investigations to validate these findings and further explore the interplay of immune modulation and viral oncogenesis in breast cancer.

## 4. Conclusions

In conclusion, the association between viral infections and breast cancer remains a compelling area for future investigation. The triple viral infections observed in our study highlight the intricate interplay between multiple viruses and their potential contribution to carcinogenesis, particularly in patients with predisposing factors such as immunosuppression, lifestyle influences, and genetic susceptibilities. These findings underscore the importance of understanding the immune-modulatory effects of viral infections in the tumor microenvironment.

To build on the hypotheses generated by this study, we propose a roadmap for future research that includes large-scale, well-controlled cohort studies to explore the prevalence and impact of viral co-infections in breast cancer. Mechanistic experiments are also needed to elucidate how these viruses interact with host cells and immune pathways to influence cancer development and progression. Furthermore, the variation in viral co-infection rates across regions underscores the necessity of collaborative international research efforts. These collaborations could standardize diagnostic protocols, enable robust comparisons across populations, and inform the development of targeted prevention and therapeutic strategies, such as vaccines or immunotherapies.

We recognize the importance of outlining actionable steps to encourage international collaborations. To address this, we propose the establishment of multinational registries for patients with viral co-infections and cancer, fostering data-sharing initiatives through consortia, and organizing interdisciplinary workshops. These efforts aim to facilitate collaborative research to better understand and address viral oncogenesis, ensuring that findings are applicable across diverse populations.

By integrating epidemiological, molecular, and clinical insights, future studies can advance our understanding of viral oncogenesis and contribute to more effective breast cancer management and prevention strategies.

## Data Availability

The data presented in this study are available from corresponding authors upon request.

## Supplementary Materials

The following supporting information can be downloaded at: https://www.mdpi.com/article/10.3390/diagnostics15010051/s1, Table S1: Overview of studies investigating viral co-infections in breast cancer (BC) across different countries. The table summarizes key characteristics, including the number of patients, average age, BC pathology, rates of viral positivity, most common co-infections, and the reported outcomes. BC: Breast cancer; DCIS: Ductal carcinoma in situ; IBC: inflammatory breast cancer; IDC: invasive ductal carcinoma; ILC: invasive lobular carcinoma; NST: Invasive breast cancer of no special type.
