# Supplementary material for: Triple Viral Infections in Advanced Breast Cancer: Insights from a Three-Case Report and Literature Review
Source: Diagnostics (Basel). 2024 Dec 28;15(1):51. doi: 10.3390/diagnostics15010051 (PMC11720534; doi:10.3390/diagnostics15010051)
Supplement: Supplementary file 1 [file diagnostics-15-00051-s001.zip › diagnostics-3337551-supplementary.pdf]

Author	Year	country	Number of patients	Avg. age (years)	Breast cancer pathology	Virus positivity (%)	Co-infection (%)	Most common viral co-infections	outcome in multiple viral infection
El-Shinawi [20]	2016	Egypt	135	51.1	IBC, and non-IBC	HPV-16 (73%), CMV (79%), EBV (41%), HSV-1 (16%), HSV-2 (17%), and HHV-8 (4%)	HPV-16/CMV/HHV-8, HPV-16/EBV/HSV-1, and CMV/EBV/HSV-1 was significantly higher in IBC tissues	CMV	Ki-67 overexpression is associated with higher chances of multiple viral infection and aggressiveness.
Al Moustafa [18]	2016	Syria	108	NA	IDC	EBV (52%), HPV (61%)	HPV and EBV (32%)	HPV	Co-presence of EBV and high-risk HPVs is associated with high-grade invasive ductal carcinoma
Naushad [22]	2017	Pakistan	250	NA	DCIS, IDC	EBV (24.4%), HPV (18.1%) and MMTV (29.3%)	HPV and EBV (9.2%), HPV and MMTV (3.2%), EBV and MMTV in (6%), HPV, EBV, and MMTV (2.4%)	MMTV	Co-infection of these viruses does not significantly correlate with the clinical outcomes of the breast cancer disease
Gupta [17]	2020	Qatar	74	55.3	IDC, NST, ILC	HPV (65%), EBV (49%)	HPV and EBV (47%)	HPV	Co-infection could be linked to a more aggressive tumor phenotype
Al Hamad [15]	2020	Jordan	100	47.2	IDC, DCIS, ILC	MMTV (11%), HPV (21%), EBV (23%)	Multiple (4.7%)	EBV	No association was found between EBV, HPV, and MMTV infections with the clinical and pathologic parameters in BC
Nagi [16]	2021	Lebanon	102	52.4	IDC, NST, ILC	HPV (65%), EBV (40%)	HPV and EBV (29%)	HPV	May play an important role in its development and/or progression of BC
Metwally [19]	2021	Egypt	80	41.3	BC	HPV (41.3%), EBV (37.5%) and HMTV (41.3%)	HPV and HMTV (25%), EBV and HMTV (2.5%), EBV and HPV (2.5%)	HPV and HMTV	No association between the co-presence of HPV, EBV, and MMTV virus and clinicopathological characteristics

Gupta [23]	2021	Croatia	72	62.4	Triple negative BC	EBV (36%), HPV (53%) and MMTV (7%)	HPV and EBV (16%). HPV, EBV, MMTV (3%)	HPV	No association between the presence/co-presence of HPVs, EBV, and MMTV virus and clinicopathological characteristics
Calderon [21]	2022	Peru	447	51.0	IDC	CMV (72.5%), HPV (2.9%)	HPV and CMV (2.2%)	CMV	No association was found between viral infections and clinicopathological findings in BC
Gupta [24]	2022	Qatar	74	NA	BC	HPV (65%), EBV (49%), MMTV (15%)	HPV and EBV (47%). HPV, EBV, and MMTV (14%)	HPV	No significant association with clinicopathological features.
Khasawneh [4]	2024	Jordan	110	55.7	BC	HPV (24%), CMV (13.6%), EBV (16.4%)	HPV and EBV (1.8%), HPV and CMV (2.7%), EBV and CMV (5.5%)	HPV	Suggest potential cooperative role of HR-HPVs and EBV oncoproteins in initiating and/or advancing various subtypes of human BC
Khasawneh [3]	2024	Jordan	103	56.0	BC	MMTV (6.8%), BLV (18.4%)	MMTV and BLV (1.9%)	BLV	No significant association with clinicopathological variables.

Table S1. Overview of studies investigating viral co-infections in breast cancer (BC) across different countries. The table summarizes key characteristics, including the number of patients, average age, BC pathology, rates of viral positivity, most common co-infections, and the reported outcomes. BC: Breast cancer; DCIS: Ductal carcinoma in situ; IBC: inflammatory breast cancer; IDC: invasive ductal carcinoma; ILC: invasive lobular carcinoma; NST: Invasive breast cancer of no special type.
